# Dentistry—Its Difficulties and Responsibilities

**Published:** 1872-12

**Authors:** H. L. Sage


### Dentistry—its Difficulties and Responsibilities.

BY H. L. SAGE, D. D. S.

*Read before, and Published by a Vote of the Connecticut. State Dental Association.*

This being the first subject on the list selected for discussion, it may be well, in the very limited time given for the preparation of a paper, to take a brief review of it.

That the practice of dentisty is attended with many and great difficulties and responsibilities, is assented to by the honest and intelligent portion of the profession.

That the difficulties only exist in the imagination, may be affirmed by the mere hireling—he who is satisfied with the ghost—the shadow—in place of the staple, tangible, permanent product of honestly applied skill.

That there are *no* responsibilities in the matter, is practically carried out by this class of dental adventurers.

In the present advanced state of the profession, with the increase of light, the influx of dental literature, the advantages of dental colleges, the improvements in instruments and appliances, there would seem to be no excuse for a neglect of any of the duties devolving upon the dentist—duties voluntarily assumed, and if not carried out, a deception and a fraud upon a confiding public—who, though not in many instances as discriminating as they might or ought to be in their choice, are no more to blame than that portion of our profession who have pandered to their ignorance—even by a profusion of gutter advertising. Then again, the rocks and fences have exhibited the artistic flourishes, by means of pot, paint and brush, of our chirographic hero of the dental art who, in this succinct and *modest* way, tells the passer-by, where to find "a good set of teeth for ten dollars."

Ignorance compounding with ignorance, the naturally low tendencies of some, with the depraved or uncultured tastes of others, and the outwardly respectable, tolerating the fumes of whisky, and other accompaning inflictions, influenced only by the paraded *semblance* of a great value, at a reduced price.

Is any one skeptical on this point? Come to a certain town I wot of, where, of the dentists, one-fifth will collectively answer the description, and of the community, a host.

Now, one of the difficulties encountered by the worthy dentist is, to overcome the bad influences exerted by these quacks. In fact, you cannot make use of their weapons, for, if you do, the recoil will come upon yourself.

Let the "shopper" go to his or her own place. All legitimate attempts to influence such, *instanter*, will be futile, except you place yourself upon the same low plane as your neighbor of the class before referred to, by adopting his tactics, and even then, you may be obliged to retire from the contest, a discomforted and a sadder, if not a wiser man.

This class of the community cannot be *instantly* converted from the error of their ways by anything like orthodox preach, ing. Convictions, require time, for their conception and birth.

How shall the hearts, the consciences, and the dormant good sense of the masses, be aroused? The most effectual plan would seem to be, by a free circulation of dental literature which shall educate them to an appreciation of their wants. to an understanding of their true interests, to a knowledge of the structure, diseases and care of the teeth and gums, and the steps necessary to be taken for their salvation; that they may understand more fully the scope of the work of the dentist, his relations to his patients, their indebtedness to him, the qualifications which should be possessed by the latter, the rules of the body of associated dentists by which he is governed, etc.

There is a large class, who, though possibly intelligent in most matters, are not sufficiently informed in regard to these.

This is one of the greatest difficulties attendant upon practice—inasmuch as it is, in a measure fostered and encouraged by unprincipled and incompetent dentists; and the respect which should only attach to true worth, is shared as well by the undeserving, or the disgrace incurred by the latter, is likewise a reflection upon the profession at large, and an obstacle in the way of dental progress.

As to checks upon the unworthy, stringent laws, will only prove effectual. If you frown upon such, the public are too apt to view it as a mere business crusade against a competitor, with the same sordid and interested motives actuating you, as him.

Stringent laws, and *education all around*, will only work the much-needed reform, leaving out of view, moral and religious qualifications.

Thus much upon this point, for hackneyed though it may be, it ought never to lose force and prominence, until the object is accomplished. And here, rests a responsibility upon every member of this society, and the time is fast approaching, when our specialty, will be held to a stricter account for the results of modes, methods and procedures than it has been in the past.

He who carelessly and needlessly exposes a pulp in the operation of preparing a cavity for filling, if not he who needlessly destroys one already exposed—he who unnecessarily extracts a valuable tooth—he who selects and fills the cavities simple and easy of access, and passes by without comment, warning, or needed attention, those requiring operations which will tax the patience, skill and strength of the operator, thus leaving the patient in blissful ignorance of the danger, and making himself responsible for their loss—he who knowingly places a bare, cold wall of metal in contact with bare pulp tissue, thus causing alveolar abscess, disfigurement and loss—violates his responsibilities. But these are only isolated examples of a class, which are of daily occurrence, and many others, will occur to the minds of most.

Ofttimes, there are difficulties in the way of avoiding these things. It involves care, effort and self-sacrifice. But "he who would save his life must first lose it."

Do we not feel, in our own experience, that the loss of a single tooth, is a calamity? A *touch* is fraught with responsibility. "Put yourself in his place," comes with a vividness and distinctness to the mind, through the natural sensibilities of every dentist that may chance to find himself in the chair, for treatment at the hand of his professional brother, which affords food for reflection for some time thereafter. And in such a case it is well, to be "made perfect through suffering."

It may modify the harshness of his operations, make his touches more gentle, his instruments sharper, with the production of pangs less keen, and enlist his sympathies in the sufferings of his patients, to such an extent, that he will *practically* admit the fact, that they are, in many instances, capable of being lessened, and the *horrors* of operative dentistry, *clothed* by the imagination with a two-fold terror, so materially reduced that the term shall be an exageration and a misnomer.

This should be the aim of every dentist.

Many organizations, frail, delicate, nervous, and extremely sensitive, though, perhaps, united to heroic and determined natures, will not without great shock and injury, go through the requisite operations, excepting great carefulness and tenderness be exercised, and proper intervals for rest and recuperation allowed, and all the means at hand employed, to make the ordeal as tolerable as possible.

Who shall say, that difficulties and responsibilities are not in such a case united, and that knowledge, skill, patience, kindness, and endurance, are not the weapons with which to meet them? A child may be so wrought upon by the experiences of the first sitting, as to render it difficult to secure his appearance again when required, and thus the loss of his teeth and permanent injury be the consequence.

One may be tempted to compromise with responsibility. For instance, a patient calls for filling, but erroneously thinking that there is no essential difference in the character of an operation, or the ability of operators, or that a clever hand at extracting teeth, *must* of necessity embody all the skill requi site for the practice of dentistry, wishes beforehand to know the price per cavity, notwithstanding, it is not, in most cases, easy to anticipate the charge, it depending upon various contingencies, such as the preliminary treatment required, the attendant difficulties and complications, the time, labor and skill necessary to be employed, etc.

A dentist of lax morals, may be tempted to chaffer with the patient, and suit the price to his or her inclination, even though he knows, that there must of necessity, be injustice *somewhere.* But the "mental reservation," comes to his relief.

Consciences with the elasticity of caoutchouc, may be very convenient for the possessors, but may operate to the disadvantage of those who may come within the range and effects of their influence.

Doctor—was heard to remark "if patients will not give me my price, I do it for less, and slight the operation. I give them their money's worth, and no more."

But has one a right to trifle with valuable organs?

He *has* a right, to decline to assume the responsibility without adequate compensation. Every dentist knows, that operations upon the natural teeth, cannot be made too perfect—and his duty to himself and his patients requires that he should not, for the sake of retaining the patronage of a picayunish person, arrange his price so that he will be obliged to adopt the course of the before mentioned individual, whose convictions of duty were not sufficiently refined to enable him to deal honestly with himself, and thus remove the temptation to inflict an injury, which would, in many cases, be the result of such a practice.

Patient, plodding, pains-taking effort should command a just and adequate compensation, in relative proportion to the intelligence and skill required, and put forth, in the treatment of any case, or the performance of any operation, to say nothing of the loss of vital force, consequent upon the faithful prosecution of dental practice.

But there is an end to responsibility, but only when all shall have been done that falls in the line of individual duty.

One is not always bound to assume a responsibility—but having done so, he must, to the extent which his ability will warrant, carry the undertaking to a successful issue. The right to assume a burden which he is not capable of carrying, is questionable. He may think his ability sufficient, or he may not. He may know to the contrary, and delude the patient into the belief, that the work in hand is faithfully performed. If he is not morally certain of his ability to achieve success, provided it be within the limits of posssibility, or that, in the event of failure, the loss would be inconsiderable, or capable of repair, he should not assume the responsibility, always provided it can be transfered to the shoulders of another, who is adequate to the emergency.

There *are* times, when, *to try*, involves, in the event of failure, no serious consequences.

There are times, when it may.

There are times, when it is the only alternative, though success may seem improbable, or uncertain. There are *discouragements* as well as difficulties. Difficulties and discouragements go hand in hand. One is consequent, though not necessarily, upon the other.

How disheartening, to have the cherished vision of permanence, dispelled by an examination of a previous operation.

Take for example, a proximal cavity in a bicuspid tooth. This may have been prepared and filled, with special care. The operation may have been faultless in every respect. The cervical borders of the cavity, may have by the delicacy of manipulation which is always requisite, escaped fracture, and a perfect and uniform adaptation and impactation of the filling may have been secured, even in this portion, where failure is the most likely to occur. Our enraptured and delighted vision, may have pronounced it the acme of excellence and stability, and yet, in a few years, a touch with the excavator, may, indicate, that the ravages of caries, have been stayed, if at all, only for a season.

These teeth, the most difficult of all to preserve, and in which the operation of filling seems to be the least efficient will sometimes baffle the skill of the most accomplished operator in this respect, especially where the teeth are so crowded as to be with difficulty cleansed, complicated, it may be, with unhealthy gums, acid or excessively alkaline secretions, and teeth incapable of resisting the agencies which combine against them, and where frequent attention is not given them by the patient, to say nothing of the deterioration which sometimes takes place in the structure of these organs.

To be, in such cases, obliged to confess that a repetition of the operation will be necessary, is one of the things, which the expert operator even, is sometimes called upon to do, owing to causes beyond his control. He can exhibit more complacency, however, when it becomes necessary to remove a filling which has been inserted by another. Most will admit that this is a rule without an exception. Though, in most cases this difficulty, would not be presented, it makes plain the duty of impressing upon the patient, the necessity there is, of *extreme care* in cleansing the proximal surfaces, which, owing to the greater difficulty of doing, in comparisons with other portions, they are, in many cases, too apt to neglect. The *how* and *wherefore* should be clearly set forth.

The last described difficulty, may be considered, so far as it goes, an argument in favor of the use of the file, according to the method of Prof. Arthur.

But most, are not yet prepared to assume the responsibility of sacrificing a considerable amount of firm, sound tooth structure, in order to render the surfaces *partially* self-cleansing, when it is admitted, that, by this, or the ordinary method of treatment, the daily attention of the patient is necessary, as well as the frequent inspection of the surfaces by the dentist, if not in the former case the repetition of the operation of filing and polishing.

But this point, it is not my present purpose to discuss.

The foregoing, may serve as an illustration of the difficulties to be encountered in dental practice. Their name is legion. The duties of the dentist are plain, and with the faithful performance thereof, his responsibility ends. But it is impossible to do justice to a subject like this, in a brief paper, and these few generalizations must suffice. Useful hints and suggestions may occur to the minds of most, which may be thrown out with profit, and thus add to, if not not complete, the review which has been made of this oft recurring theme.

